# Supplementary material for: Chromothripsis during telomere crisis is independent of NHEJ, and consistent with a replicative origin
Source: Genome Res. 2019 May;29(5):737–49. doi: 10.1101/gr.240705.118 (PMC6499312; doi:10.1101/gr.240705.118)
Supplement: Supplemental Material [file supp_gr.240705.118_Supplemental_file_1.zip › contigs/annotated_contigs/DB51/contig.2.DB51_length_553_mean_cov_8.47920433996.docx]

**DB51_length_553_mean_cov_8.47920433996**

```
ACTGGCCTTCATGTGCATAAATGGCCCTAAGGTGTACAGCTGACCTGATGCATATAGCTGACCCCATGCATATAGTTGACCCGATGCAT
 >chr6:164348841-164349050 - E=5e-90 p=0e+00
ATAGCTGACCCTGATGCATATAGCTGACCCTGATGCATATAGCTGACCCTAATGCATATAGCTGACCTGATGCATATAGCTGACCTGAT

GCATATAGCCGACCCTGATGCATATAGCTGA|CGCTGATGCATATAGCTGAGCCTAT|GCTAACACAGTGAAATCCCGTCTCCACTAAA
                                                         >chr6:158067452-158067754 + E=
AATACAAAAAAATGAGCCGGGTGTGGTGGCAGGCGCTTGTAATCCCAGCTATTCAGGAGGCTGAGACCAGAGAATCGCTTGAACCCAGG
3e-144
AGGCAGAGGTTGCAGTGAGCTGAGATAGCGCCACTGCACTCCAGCCTGGGTGACAGAGCGACACTCCATCTCAAAAAAAAAAAAGAGGA

AACAAAATGTTAGAGAATGTTTCCAGAGCAGCGTGGTCCTCTTTGCTCTTTATAATTAAATTTTTATAGAAGTGTCTATCTACACTT|T

AAATAAAATTCTGAAGATAGGT
```
